# Acute abdomen caused by a large solitary jejunal diverticulum that induced a midgut volvulus. Report of a case

**DOI:** 10.1016/j.ijscr.2020.07.082

**Published:** 2020-08-12

**Authors:** Aradhya Nigam, Faye F. Gao, Mark A. Steves, Paul H. Sugarbaker

**Affiliations:** a Department of Surgery, MedStar Washington Hospital Center, Washington, DC, USA; b Department of Pathology, MedStar Washington Hospital Center, Washington, DC, USA

**Keywords:** Pulsion diverticulum, Acute abdomen, Midgut volvulus, Case report

## Abstract

**Background:**

Jejunal diverticula are a rare subtype of false diverticula found in the gastrointestinal tract. When present, they are usually multiple, in the proximal jejunum and asymptomatic. Rarely, they can cause acute complications that can develop into an acute abdomen requiring surgical intervention. We present the rare manifestation of a single jejunal diverticulum causing midgut volvulus and bowel ischemia. Early surgical intervention with small bowel derotation allowed complete recovery.

**Methods:**

Data regarding the case, operative intervention, and patient follow-up was prospectively accumulated with permission of the patient at an academic institution. All patient identifiers were removed. All research steps were performed under guidance outlined in the SCARE criteria.

**Results:**

We present the case of a 78-year-old man who presented to our institution with an acute abdomen. CT imaging demonstrated a whirlpool sign without an obvious lead point. The patient was without prior surgical intervention. Urgent exploratory laparotomy revealed a midgut volvulus with associated bowel ischemia and impending infarction secondary to adhesive disease from a large single jejunal diverticulum. Derotation resulted in gradual recovery of bowel vascularity. Resection of the portion of the jejunum containing the diverticulum resulted in full clinical recovery of the patient.

**Conclusions:**

Complications of jejunal diverticula, although rare, should be considered as part of the differential diagnosis of an acute abdomen of unknown etiology. Urgent surgical intervention was required to avoid a potential catastrophic outcome.

## Highlights

• Diverticuli are found throughout the gastrointestinal tract from esophagus to rectum.
• In the jejunum diverticuli are most commonly proximal, multiple and asymptomatic.
• A large solitary jejunal diverticulum caused life endangering midgut volvulus.
• In the absence of timely surgical intervention the condition would have been fatal.

## 1 Introduction

Diverticuli of the gastrointestinal tract may occur from the upper esophagus down to the rectum. The outpouchings of the gastrointestinal tract may be true diverticuli that involve all layers of the bowel wall. The most common true diverticulum is a Meckel’s diverticulum present in approximately 2% of the population and usually found in the ileum [1]. Alternatively, the outpouching can be of mucosa and submucosa devoid of the usual smooth muscle layer referred to as false diverticuli. A false diverticulum of the small or large bowel usually occurs at the mesenteric border of the bowel. The outpouching is in intimate association with the site where arterioles penetrate the muscular wall of the bowel [[2], [3], [4]].

Different locations in the GI tract predispose to true and false diverticuli. Diverticuli are common in the esophagus but are much less common in the stomach [5,6]. They are common in the duodenum and at autopsy series may be present in 10–20% of persons [7]. Jejunal and ileal diverticuli are much less common occurring in less than 1% of the population [8,9]. Then, in the colon false diverticuli become extremely common. It is estimated that approximately 75% of Americans over the age of 80 will have diverticuli associated with the colon [10].

Jejunal diverticuli are uncommon and when they do exist are usually asymptomatic [11,12]. The symptoms that they cause may be incomplete bowel obstruction, acute inflammation, hemorrhage or malabsorption from bacterial overgrowth within the diverticulum in up to 10% of cases [[13], [14], [15]]. In this manuscript we report on a large solitary jejunal diverticulum that caused an acute abdomen as a result of a small bowel volvulus. The volvulus resulted in obstruction of the venous outflow, intense abdominal pain and required emergency surgery. This manuscript describes the pathology associated with the causation of an acute abdomen and speculates as to how the solitary diverticulum induced a life-endangering volvulus of the small bowel and its mesentery.

## 2 Materials and methods

Data on this patient was prospectively accumulated at an academic institution. This research work has been reported in line with the SCARE criteria [16]. This study was registered as a case report on the www.researchregistry.com website with UIN 5776.

## 3 Case report

**Fig. 1:**
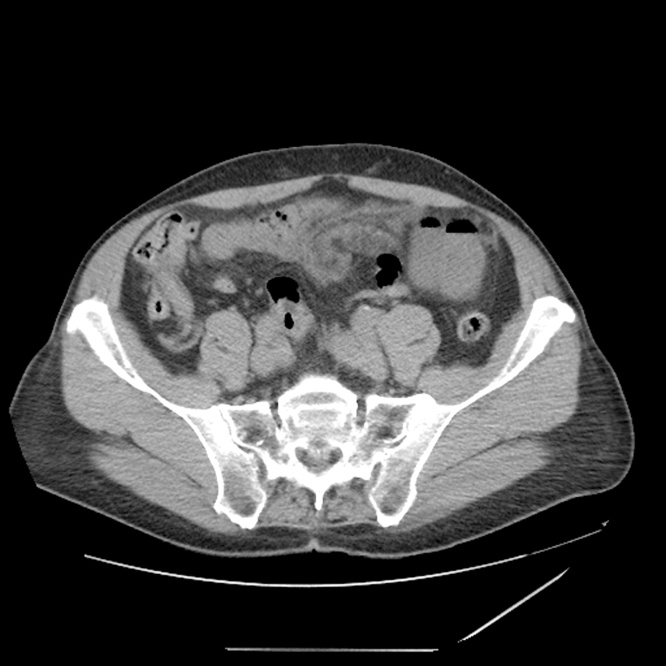
CT of the abdomen in a 78-year-old man with increasing abdominal pain over approximately 6 h. The CT scan shows whirling of the mid-abdominal mesentery with edematous changes of the fat and congestive prominence of mesenteric vessels amongst small bowel loops at this level. Early small bowel obstruction was seen with transition point in the left lower abdomen.

A previously well 78-year-old male in the mid-morning of April 5, 2020 developed increasing abdominal pain. The pain was steadily increasing over 6 h which prompted urgent travel to an emergency room. The pain was treated with intravenous morphine. A CT with intravenous contrast was performed. It showed a “whirling” of the small bowel mesentery with edematous changes concerning for volvulus (Fig. 1). A CT with oral contrast followed. Although a large amount of oral contrast was given, it remained sequestered in the stomach and did not pass into the duodenum.

**Fig. 2:**
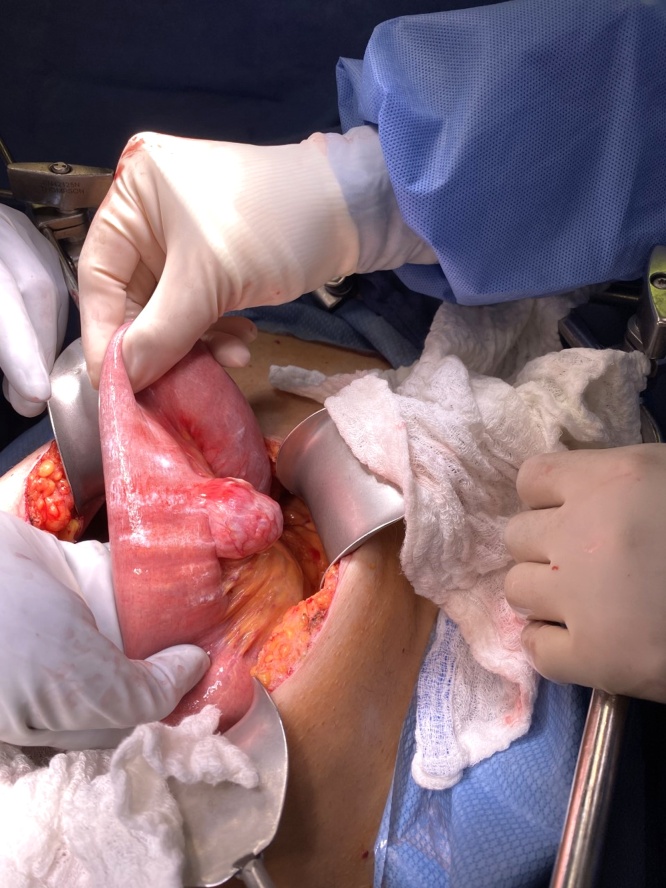
Jejunal diverticulum seen at the time of exploratory laparotomy prior to resection. The outpouching of the bowel wall occurs at its mesenteric border.

The patient’s vital signs were stable, yet due to unrelenting pain, he was taken urgently to the operating room for exploratory laparotomy. Upon opening the abdomen, the small bowel was profoundly discolored from venous congestion. The bowel was twisted on its mesentery causing obstruction of venous outflow. There was marked fibrin deposition throughout the abdomen with a large amount of inflammatory exudate. A large solitary jejunal diverticulum measuring approximately 5 cm in length was evident approximately 45 cm from the duodenojejunal junction. The apex of the diverticulum was adherent to the abdominal wall. Pictures of the solitary jejunal diverticulum in-situ were taken (Fig. 2). The diverticulum appeared to be inflamed with possible impending perforation. No enteric material was noted in the area of the jejunal diverticulum.

**Fig. 3:**
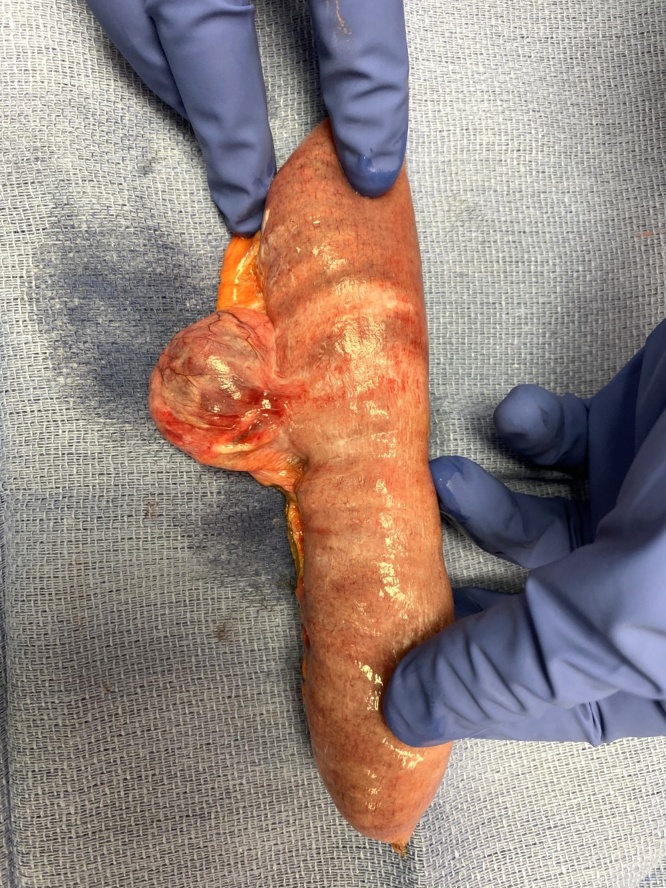
Resected specimen of the jejunal diverticulum. The diverticulum was removed with approximately 15 cm of bowel on each side of the diverticulum.

After derotation of the bowel, the abdomen was copiously irrigated with warm saline. Impending infarction resolved after approximately twenty minutes. The normal color returned to the small bowel and fully restored after forty minutes. A generous resection of the diverticulum including an approximate 30 cm segment of the jejunum was performed (Fig. 3). A stapled side-to-side anastomosis was completed. Additional large volume irrigation of the abdomen and pelvis was performed to remove as much exudate possible. The abdomen was closed in a routine fashion.

The patient recovered from surgery without incident. He was maintained on nasogastric suctioning for 72 h initially with a large volume of bile-stained contents from the stomach. As the volume of nasogastric suctioning reduced, the tube was removed. The patient gradually resumed sufficient gastrointestinal function to be discharged from the hospital on his sixth postoperative day. Normal alimentation occurred after approximately eight weeks.

**Fig. 4:**
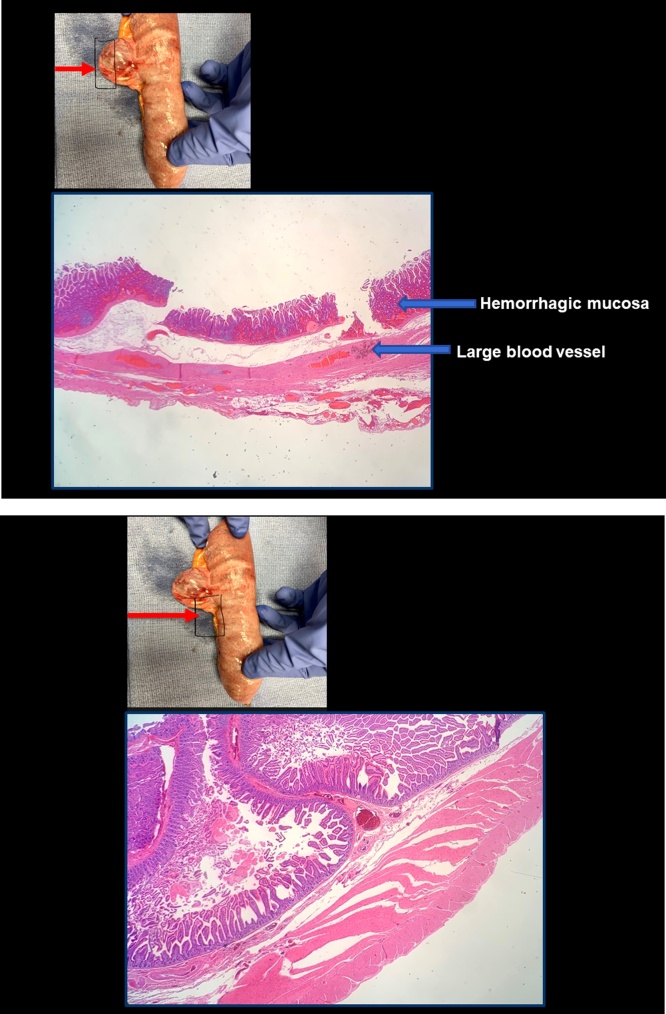
(top). Histopathologic sections through apex of the false diverticulum. The wall is composed of mucosa, muscularis mucosa and submucosa. The muscularis mucosa and submucosa show large dilated blood vessels. The muscularis propria, both circular and longitudinal layers are absent. Fig. 4 (bottom). Histopathologic section through the small bowel adjacent to but not within the diverticulum. All layers of the jejunal wall are intact including muscularis propria (Hematoxylin and eosin, 2×).

Pathologic examination of the specimen determined that the normal muscular coating of the bowel was absent in the area of the diverticulum. This confirmed that this was a false diverticulum where mucosa and submucosa were present in the outpouching in the absence of a surrounding layer of smooth muscle (Fig. 4).

## 4 Discussion

Jejunal diverticuli may be small or large. They may be solitary or multiple. The causes for surgical removal of a jejunal diverticulum are complete or incomplete bowel obstruction, acute inflammation, hemorrhage, or malabsorption from bacterial overgrowth within the diverticulum [13,17,18]. Small bowel volvulus with obstruction of the venous outflow should be added to the list of indications for surgical intervention. In this patient we hypothesize that the diverticulum had become inflamed and caused a localized peritonitis prior to an impending perforation. This disruption of the normal peritoneal surface resulted in small bowel becoming fixed to the abdominal wall and then twisting on itself to result in the volvulus. An exact cause and effect relationship between the jejunal diverticulum and the extensive volvulus of the small bowel with obstruction of venous outflow is not completely clear.

Critical to this patient’s management was the decision to proceed to urgent exploratory laparotomy without an extended period of observation. The patient did have intense pain and abdominal symptoms. The CT scan on this patient showed whirling of the mesentery which indicates profound compromise of the bowel and a need for immediate surgical intervention [19]. A delay in performing the exploratory laparotomy would have been catastrophic. Mechanical derotation of the bowel back to its normal position allowed it to recover from its venous compromise. An extended delay in proceeding to laparotomy would have resulted in bowel infarction and loss of a major portion of the jejunum and at least some of the ileum.

The treatment indicated for a complication secondary to a jejunal diverticulum is almost always resection of the involved portion of the bowel followed by a small bowel anastomosis. In some instances, there will be multiple jejunal diverticuli and a large portion of the small bowel is involved by this pathology. In this situation the portion of the jejunum causing symptoms and endangering bowel function should be resected without performing very extensive bowel resections [20]. In this particular patient, the jejunal diverticulum was solitary and the decision to resect and perform and anastomosis was straightforward. Care must be taken to carefully inspect the bowel because the diverticulum may be within the mesentery of the small bowel and not immediately apparent.

In this instance the pathologist did confirm that this was a false diverticulum with the muscularis propria absent from the wall of the diverticulum. The causation of such a “false diverticulum” is not clear except that the defect is almost always on the mesenteric side of the bowel and the outpouching directly associated with arteriolar penetration of the bowel wall at its mesenteric border. As the size of the diverticulum grows, the pressure on its wall increases by the law of Laplace. As the diverticulum reaches the size seen in this patient it is not surprising that vascular compromise and impending necrosis with perforation of the distal portion of diverticulum may occur.

A review of the different types and locations of diverticuli that occur throughout the gastrointestinal tract is presented in Table 1. False diverticuli are present at multiple sites within the gastrointestinal tract. True diverticuli are less common and include traction diverticuli in the esophagus and Meckel’s diverticuli in the distal ileum. The frequency of a surgical intervention required for diverticuli is difficult to determine and has not been documented in the literature. However, acute complications of diverticuli often require urgent surgical intervention and may only be diagnosed at the time of surgery, as in this patient. Although this remains the second case to describe a solitary jejunal diverticulum resulting in midgut volvulus, it is the first to describe a resultant venous congestion as a complication requiring urgent intervention [19].

**Table 1:** Diverticuli of the gastrointestinal tract.

Site	Type	Location	Incidence	Pathogenesis	Symptoms/signs prior to surgical intervention
Esophagus	FALSE	Top and bottom	1%	Pulsion	Dysphagia
	TRUE	Middle		Traction	
Stomach	TRUE	Posterior wall	0.04%	Unknown	Fullness
Duodenum	FALSE	2^nd^ part of duodenum	Up to 20%	Unknown	Early satiety
					Bleeding
					Perforation
Jejunum/Ileum	TRUE	Meckel’s diverticulum Terminal ileum	2%	Congenital	Perforation
					Volvulus
	FALSE	More common in ileum	1%	Unknown	Bleeding
					Perforation
					Volvulus
Large bowel	FALSE	Most common in sigmoid colon	50%	Increases with age	Bleeding
					Perforation
					Acute/chronic infection

## Declaration of Competing Interest

Aradhya Nigam, Faye F. Gao, Mark A. Steves, Paul H. Sugarbaker have no conflicts of interest to declare.

## Funding

Data management and secretarial support provided by Foundation for Applied Research in Gastrointestinal Oncology.

## Ethical approval

Local IRB-approval for this case report was not required:

MedStar Health Institutional Review Board has determined that a case report of less than three (3) patients does not meet the DHHS definition of research (45 CFR 46.102(d)(pre-2018)/45 CFR 46.102(l)(1/19/2017)) or the FDA definition of clinical investigation (21 CFR 46.102(c)) and therefore are not subject to IRB review requirements and do not require IRB approval.

This case report is of 1 patient.

## Consent

Written and signed consent was obtained from the patient.

## Author contribution

Paul H. Sugarbaker: study concept or design, data collection, data analysis or interpretation, writing the paper.

Aradhya Nigam: data collection, data analysis or interpretation, writing the paper.

Faye F. Gao: study concept or design, data collection, data analysis or interpretation, writing the paper.

Mark A. Steves: data collection, data analysis or interpretation.

## Registration of research studies

This study was registered as a case report on the www.researchregistry.com website with UIN 5776.

## Guarantor

Paul H. Sugarbaker, MD.

## Provenance and peer review

Not commissioned, externally peer-reviewed.
